# Association of hearing loss and risk of depression: a systematic review and meta-analysis

**DOI:** 10.3389/fneur.2024.1446262

**Published:** 2024-10-21

**Authors:** Jingxuan Wei, Yang Li, Xiongbin Gui

**Affiliations:** ^1^Graduate School, Guangxi University of Chinese Medicine, Nanning, China; ^2^School of Nursing, Guangxi University of Chinese Medicine, Nanning, China; ^3^The First Affiliated Hospital of Guangxi University of Chinese Medicine, Nanning, China

**Keywords:** hearing loss, depression, meta-analysis, sudden sensorineural hearing loss, age group, follow-up years

## Abstract

**Background:**

Previous studies have suggested a possible link between hearing loss and the risk of depression. However, the strength of this association remains uncertain across varying follow-up periods, age groups, cohort studies, and longitudinal study designs.

**Methods:**

We conducted an extensive literature search across PubMed, Embase, and the Cochrane Library databases to retrieve pertinent studies. The quality of observational studies was assessed using the Newcastle–Ottawa Scale. Synthesis and meta-analysis of odds ratios (ORs) along with corresponding 95% confidence intervals (CIs) were performed using Stata 14.0. Funnel plot analysis and Egger’s regression test were utilized to assess potential publication bias.

**Results:**

This meta-analysis comprised 24 cohort studies conducted between 2007 and 2024, with sample sizes ranging from 548 to 254,466 participants. Among these participants, 24,304 had experienced depression events. The pooling analysis shows that hearing loss is associated with an increased risk of depression (OR = 1.35; 95%CI: 1.27–1.44). In the subgroup analysis, the retrospective cohort exhibited a slightly higher risk of depression compared to the prospective cohort (OR = 1.43; 95% CI: 1.30–1.58). There are differences in the risk of depression among young, middle-aged, and older individuals, with older adults facing a higher risk (OR = 1.33, 95% CI: 1.21–1.45). Additionally, the risk of depression was slightly higher in the sudden sensorineural hearing loss (SSNHL) group compared to the non-SSNHL group (OR = 1.62; 95% CI: 1.27–2.07). Furthermore, in cohorts with a follow-up time ≥ 5 years, the risk of depression was higher compared to those with <5 years of follow-up (OR = 1.39; 95% CI: 1.26–1.54).

**Conclusion:**

Our meta-analysis shows that hearing loss increases the risk of depression. These findings provide evidence that hearing loss should be recognized as an independent risk factor for depression.

**Clinical trial registration:**

https://www.crd.york.ac.uk/PROSPERO/, identifier: CRD42024502043.

## 1 Introduction

Depression, a mental disorder, significantly impacts an individual’s quality of life, presenting as negative emotions, thoughts, and behaviors. It impairs social, occupational, and educational functioning, elevating the risk of suicide and mortality (1). Globally, it is the leading cause of disability, affecting around 280 million people (2, 3). The prevalence of depression among the older adults amplifies the risk of suicidal tendencies (4). While past studies have predominantly centered on depression in older adults, overlooking its impact on youth and children (5), it is crucial to acknowledge its significance. Depression emerges as the primary cause of illness and disability in younger age groups, with recent data indicating that half of all mental illnesses in adulthood originate from issues occurring before the age of 14 (6). Despite heightened societal attention toward depression prevention in recent years, outcomes remain unsatisfactory due to the complexity of the condition and its multifactorial etiology (7–9). Hearing loss may represent a significant risk factor for the occurrence of depression (10–13).

To date, observations have linked hearing loss (HL), including conductive, sensorineural, severe sensorineural, and mixed hearing loss, to the development of depression, making HL a significant global public health concern (14). It currently ranks as the fourth leading cause of disability worldwide. Early-onset hearing loss can detrimentally impact children’s speech development (15), while adults with hearing loss often experience a profound sense of isolation, leading to social and familial withdrawal (16). HL can result in reduced neural activation triggered by auditory stimuli, leading to compensatory activation of perceptual control networks (17). This burden on the neural system impairs the processing of emotional information, a common occurrence in older adults with depression (18). Existing research predominantly concentrates on elucidating the mechanisms linking hearing impairment to late-life depression, yet there is a dearth of observation concerning individuals with extensive hearing loss. Lawrence et al.’s meta-analysis exclusively targeted the older adults, lacking observations from other age groups and longitudinal follow-up analysis (19). Similarly, Zhang et al.’s meta-analysis solely addressed sensorineural hearing loss and included various study designs, leading to increased outcome heterogeneity (20). Given the significance of this matter, the constraints of the previous review, and the emergence of fresh evidence, we conducted a systematic review and meta-analysis based on cohort studies to assess the correlation between HL and the risk of depression.

## 2 Methods

This study adhered to the Preferred Reporting Items for Systematic Reviews and Meta-Analyses (PRISMA) guidelines (21). The protocol for this meta-analysis was registered with the International Prospective Register of Systematic Reviews under registration number CRD42024502043.

### 2.1 Data sources

To identify studies investigating the potential associations between hearing loss and the risk of depression, we systematically searched three electronic databases (PubMed, Embase, and Cochrane Library) for studies published up to August 29, 2024. Furthermore, we manually reviewed the reference lists of relevant review articles for additional reports.

The search strategy involved two themes, delineating various types of hearing loss as exposure variables, while considering depression risk as the outcome. To enhance the comprehensiveness of the literature search, all forms of hearing impairment, anxiety, and depression states were considered, with no geographic restrictions applied. The search string for hearing loss included: “Hearing Loss [Mesh],” “Hearing Loss*,” “Hypoacus*,” “Hearing Impairment*,” and “Transitory Deafness*.” For depression, the terms used were: “Depression [Mesh],” “Anxiety [Mesh],” “Depressive Symptom,” “Emotional Depression*,” “Anxiet*,” and “Anxiousness.” The full search strategy for these databases is provided in Supplementary Tables S1–S3.

### 2.2 Eligibility criteria

The present study included original literature that met the following inclusion criteria: (1) Patients with hearing loss, whose exposure was confirmed through self-reported questionnaires, pure-tone audiometry tests (PTA), insurance claims records, and hospital diagnostic records. (2) Investigations of the association between hearing loss and the risk of incident depression. In this meta-analysis, “any hearing loss” was defined as “individuals who experienced any type of hearing loss previously,” with depression selected as the primary outcome and anxiety as the secondary outcome. (3) Anxiety and depression were identified through anxiety and depression scales, insurance claims records, hospital diagnoses, and self-reporting. (4) Type of study: cohort studies (prospective, retrospective, and mixed prospective-retrospective cohort studies). (5) Studies included in the analysis provided risk estimates, such as odds ratios (ORs), risk ratios (RRs), hazard ratios (HRs) with 95% confidence intervals (95% CIs), or sufficient data for calculation.

Exclusion criteria were as follows: (1) Case studies, reports, reviews, conference abstracts. (2) Studies lacking follow-up data on depression beyond baseline measurements. (3) Among the outcome measures, only data on anxiety were available; however, there were no studies addressing depression. If more than one study reported data from the same cohort, we included the study with the longest follow-up or the largest number of participants.

### 2.3 Study selection

Two researchers (WJX and LY) conducted initial screening by reviewing study titles and abstracts, followed by downloading and reviewing the full texts of potentially eligible studies to ascertain final inclusion based on eligibility criteria. In cases of disagreement between the two researchers, decisions were reached through discussion and consultation with a third researcher (GXB).

### 2.4 Data extraction

Two researchers (WJX and LY) independently collected information using a pre-designed checklist. In cases of disagreement, final decisions were made by researchers (GXB) after a comprehensive review of the full text. The checklist aimed to gather detailed information, such as publication details (first author, publication year, country), study characteristics (study type, follow-up duration, average age/age range, number of cases, control group), exposure and outcome diagnostic criteria, subgroup stratification (including patient age, follow-up time, study type, type of hearing impairment, and duration of follow-up), adjustment for confounding factors, and corresponding 95% confidence interval effect sizes (selecting effect sizes adjusted for all available covariates). Additionally, to ensure data accuracy, patients reporting depression at baseline were excluded, and statistical results were prioritized accordingly.

### 2.5 Risk of bias

Two independent researchers (WJX and GXB) employed the Newcastle-Ottawa Scale (NOS; Available from: http://www.ohri.ca/programs/clinical_epidemiology/oxford.asp) to evaluate the quality of the studies, categorizing them into three levels: low risk of bias (7 or above), medium risk of bias (4–6), and high risk of bias (3 or below).

### 2.6 Statistical analysis

Pooled odds ratios (ORs) and their corresponding 95% confidence intervals (CIs) were calculated as reliable measures of depression risk, regardless of study design. Given the low incidence of depression in the population, ORs were considered interchangeable with relative risks (RRs) and hazard ratios (HRs) in this meta-analysis (22). In instances of significant heterogeneity (*p* < 0.1 or *I*^2^ > 50%), the random-effects model was applied; otherwise, a fixed-effects model was utilized. Sensitivity analyses were conducted to assess the robustness of our pooled results and to evaluate the influence of individual studies on the overall estimates. Publication bias was evaluated using funnel plots and Egger’s test. All statistical analyses were performed using Stata software, version 14.0.

## 3 Results

### 3.1 Study selection

**Figure 1:**
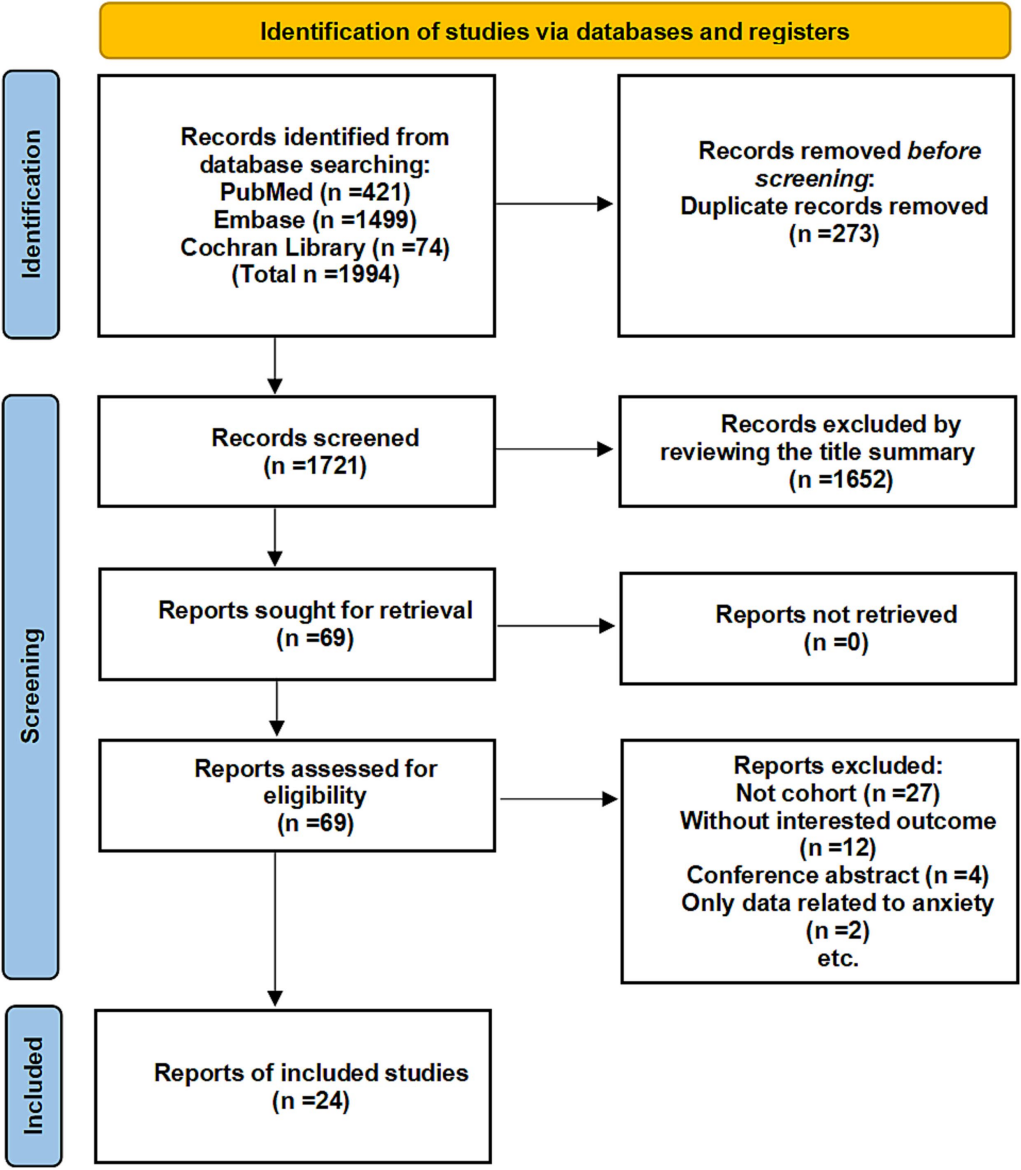
Flow diagram of the search strategy and study selection.

A total of 1,994 records were obtained from electronic databases. Following the removal of duplicates through automated tools (NoteExpress) and manual searches, 1,721 articles underwent initial screening. Among these, 1,652 articles were excluded due to unrelated participant demographics or outcome data. Ultimately, after reviewing the full-text of the remaining articles, 24 studies (10–13, 23–42) met the eligibility criteria (Figure 1).

### 3.2 Study characteristics

The 24 studies, spanning from 2007 to 2024, encompassed sample sizes ranging from 548 to 254,466 participants, of whom 24,304 had experienced depression events. Participants were followed for periods ranging from 1 to 25 years. All 24 articles were cohort studies, comprising 14 prospective cohort studies, 9 retrospective cohort studies, and 1 mixed prospective-retrospective design. The majority of these studies were conducted in Europe and Asia, with four originating from the United States. The assessment of hearing ability predominantly relied on self-reporting, pure tone audiometry, and classification according to the International Classification of Diseases (ICD-10). Diagnosis of depression was confirmed and categorized using various methods, including insurance claims registries, medical records, depression or anxiety Scales, and self-reported anxiety symptoms. The characteristics of 24 cohort studies are summarized in Table 1.

**Table 1:** Basic characteristics of the included studies.

Author	Year	Country	Study type	Sample size	follow-up time (years)	Case number	Hearing loss type	Depression type	Confounders adjusted	Age	NOS scores
A. Yu et al.	2019	United Kingdom	Retrospective cohort study	3,931	4 years	188	Hearing loss	Depression	Age, sex, cohabitation, education, wealth, physical activity, smoking, BMI and chronic illness, mobility limitations and cognition	64 ± 8.4 years	7
Adam Simning et al.	2019	United States	Prospective cohort study	7,505	1 year	387	Hearing loss	Depression	Age, sex, marital status, race/ethnicity, education, social contacts, medical conditions, activities of daily living, instrumental activities of daily living	>65 years	6
Chih-Chieh Tseng et al.	2016	Taiwan, China	Retrospective cohort Study	8,585	8 years	51	Sudden sensorineural hearing loss	Depression	Age, sex, common comorbidities (including hypertension, diabetes mellitus, dyslipidemia, coronary artery disease, congestive heart failure, hyperthyroidism, hypothyroidism, cerebrovascular disease, and malignancy), urbanization and monthly income	51 years (interquartile rang)	7
Chuan-Ming Li et al.	2014	United States	Prospective cohort study	18,318	4 years	3,649	Hearing loss	Depression	Age, sex, race/ethnicity, marriage, living alone, educational level, income, health insurance, body mass index, smoking, binge drinking, self-reported health status, cardiovascular disease, cancer, diabetes mellitus, hypertension, sleep disorder, and trouble seeing	≥18 years	7
Emma Butcher et al.	2022	United Kingdom	Prospective cohort study	10,858	14 years	3,003	Hearing loss	Depression	Sampling design and attrition, and include imputed data for missing values (0 for sex and ethnic background, 2 for parental education, 32 for maternal age, 44 for NICU, 2217 for LLI, and 3,571 for HL)	Ages 9 months, 3, 5, 7, 11, and 14 years	6
Hélène Amieva et al.	2018	French	Prospective cohort study	3,080	25 years	181	Hearing loss	Depression	Age, diabetes, hypertension, myocardial infarction, cerebrovascular disease, obesity, depression, income tertile, and education	75.3 ± 6.8 years	7
Hideyuki Saito et al.	2010	Japan	Prospective cohort study	548	2 years	22	Age-related hearing loss	Depression	Age, sex, education, living circum-stances, lifestyle factors, history of illnesses, vision impairment, objective hearing loss	≥60 years	8
Hye Jun Kim et al.	2023	Korea	Prospective cohort study and retrospective cohort study	254,466	16 years	491	Hearing loss	Depression	Age, sex, household income, body mass index, type 2 diabetes mellitus, hypertension, dyslipidemia, and smoking	69.5 ± 9.8 years and 54.5 ± 6.5 years	8
Jae Woo Choi et al.	2021	Korea	Retrospective cohort study	14,212	12 years	3,553	Hearing loss	Depression	Sex, age, index year, comorbidities, history of depression, household income, insurance type, and residential area	≥20 years	8
Jong-Yeup Kim et al.	2018	Korea	Retrospective cohort study	8,850	11 years	225	Idiopathic sudden sensorineural hearing loss	Depression	Age, sex, residential area, household income, and comorbidities	<45 years, 45–64 years, >64 years	7
Katharine K. Brewster	2018	United States	Prospective cohort study	1,204	10 years	180	Hearing Impairment	Depression	Age, race, gender, education	70–79	7
Kee-Lee Chou et al.	2008	United Kingdom	Prospective cohort study	3,782	2 years	1,198	Hearing loss	Depression	Age, sex, marital status, education, employment, income, illnesses, physical impairment, lifestyle factors, family support	>65 years	8
Marieke Pronk et al.	2011	Netherlands	Prospective cohort study	3,107	1 year	1,821	Hearing loss	Depression	Education, income, self-reported vision, diseases, cognition	74.50 years (Mean age)	6
Marijke Boorsma et al.	2012	Netherlands	Retrospective cohort Study	3,047	2 years	1,486 and 947	Hearing loss	Depression	None	≥60 years	5
Olivia J. Killeen et al.	2022	United States	Prospective cohort study	7,593	8 years		Hearing loss	Depression	Sex, race/ethnicity, and highest education, age, dementia status, number of chronic medical conditions, Medicaid eligibility, proxy respondent, and survey round	≥65 years	8
Quentin Lisan et al.	2019	France	Prospective cohort study	7,591	6 years	479	Hearing loss	Depression	Age, sex, physical activity, body mass index, diabetes, hypertension, prevalent cardiovascular disease, alcohol consumption, smoking status, education level, and living alone	59.8 ± 6.3 years	9
So Young Kim et al.	2020	Korea	Retrospective cohort study	21,640	4 years	332	Sudden sensorineural hearing loss	Depression	Age, income, and region of residence were classified as previous studies	20–80 years	7
So Young Kim et al.	2017	Korea	Retrospective cohort study	30,680	11 years	487	Hearing loss	Depression	Age, sex, income, region of residence, dementia, hypertension, diabetes, and dyslipidemia.	All	8
Suzanne Cosh et al.	2018	French	Prospective cohort study	8,344	12 years	1,484	Hearing loss	(1) Major depressive disorder (2) Depression symptoms	Sex, age, center, education, income, marital status, psychotropic medication use, MMSE, functional ability, falls, BMI, hypertension, diabetes, smoking, alcohol consumption, and history of stroke and myocardial infer citron	74.2 ± 5.5 years	9
Wei-Ting Hsu et al.	2016	Taiwan, China	Retrospective cohort study	25,215	12 years	273	Acquired sensory hearing loss	Depression	Age, sex, and comorbidities of cirrhosis, hypertension, hyperlipidemia, diabetes mellitus, asthma, chronic kidney disease, coronary artery disease, alcohol-related illness, anxiety, COPD and stroke, and medication of steroid	All	7
Wenwen Liu et al.	2022	China	Prospective cohort study	13,690	4 years	1,231	Hearing loss	Depression	Age, sex, education, area of residence, marital status, household income, smoking, drinking, BMI, hypertension, diabetes, and CVD	58.7 ± 9.4 years	8
Xueying Li et al.	2024	China	Retrospective cohort study	10,050	3 years	875	Hearing loss	Depression	Demographic characteristics, socioeconomic status, lifestyle, and health-related factors	58.9 ± 9.2 years	8
Yun-Guang Liu et al.	2022	China	Prospective cohort study	9,492	7 years	363	Hearing loss	Depression	Age, sex, residence, marital status, smoking history, alcohol consumption history, hypertension, diabetes, heart disease, digestive disease, and arthritis; wearing glasses and hearing aids	58.12 ± 9.0 years	8
Zhihong Lu et al.	2024	China	Prospective cohort study	12,333		1,398	Hearing loss	Depression	Age, hukou, gender, marital status, income, educational attainment, smoking, and drinking were added	60–80 years	6

BMI, body mass index; COPD, chronic obstructive pulmonary disease; CVD, cardiovascular disease; HL, hearing loss; LLI, limb loss indicator; MMSE, mini-mental state examination; NICU, neonatal intensive care unit.

### 3.3 Quality assessment

According to Newcastle–Ottawa Scale, the mean score of included studies was 7.00 ± 0.95, with a media of 7. Nineteen of the studies were judged as having a low risk of bias and four as having a moderate risk, indicating that the methodological quality of the included studies was medium or high.

### 3.4 Any hearing loss and depression risk

**Figure 2:**
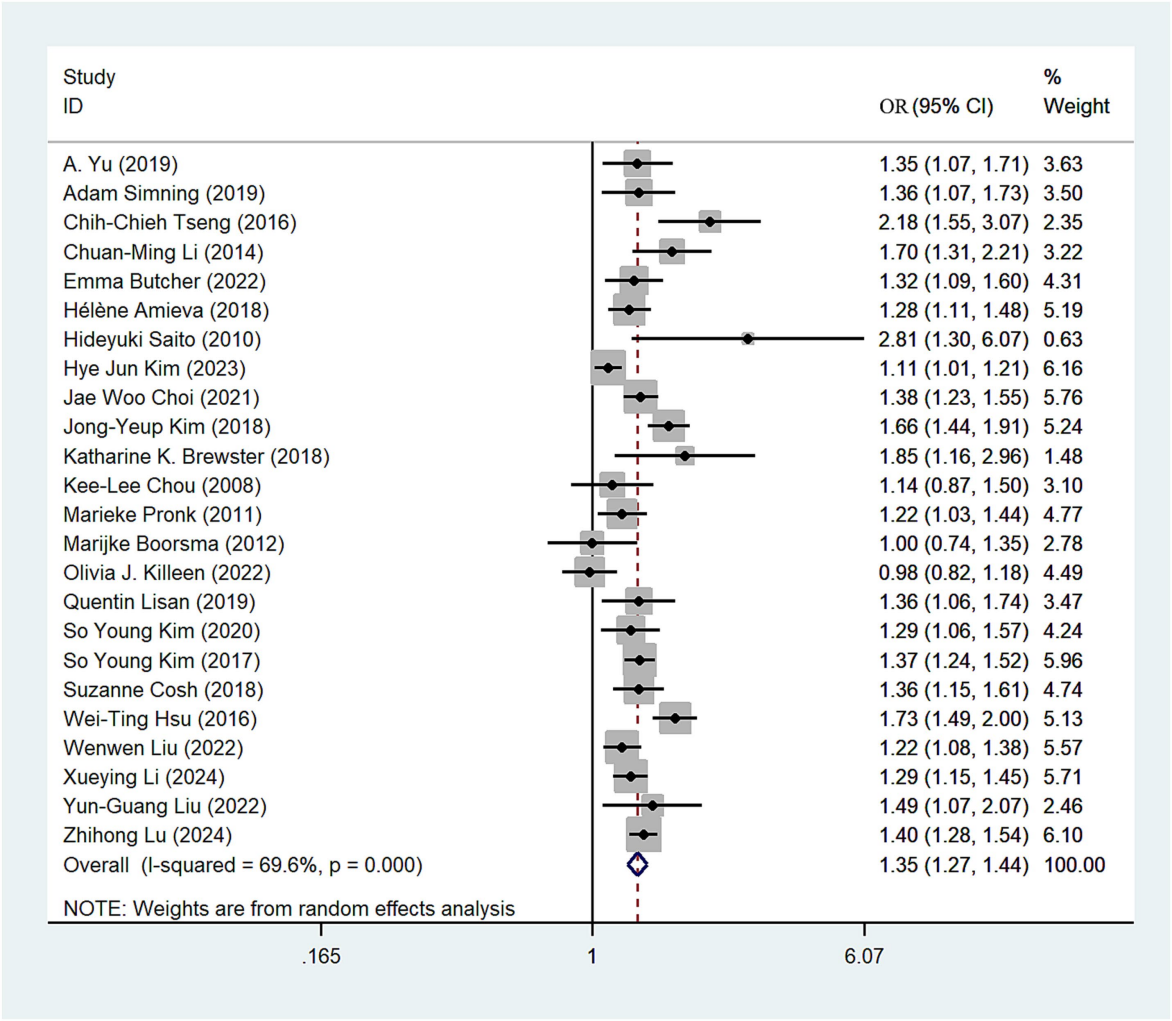
Forest plot of odds ratios of total depression for hearing loss participants.

Twenty-four (10–13, 23–42) studies assessed the relationship between any hearing loss and the risk of depression. The OR for hearing loss associated with the risk of depression was 1.35 in a random-effects model (OR = 1.35; 95%CI: 1.27–1.44, *I*^2^ = 69.6%; Figure 2). There was high heterogeneity among the literature selected for this study. We performed a sensitivity analysis. Sensitivity analysis revealed some degree of heterogeneity, but no individual study reversed the magnitude of the combined effect, indicating the robustness of the results (Supplementary Figure S1).

### 3.5 Subgroup analysis

#### 3.5.1 Study type

**Figure 3:**
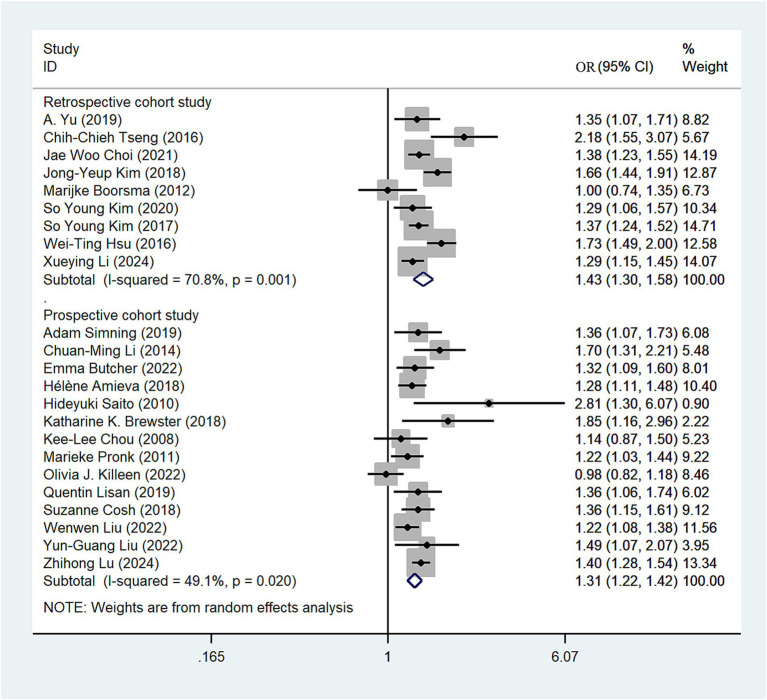
Forest plot of subgroup analysis by study type.

We conducted subgroup analyses based on study type, revealing a nearly equivalent risk of depression among hearing loss patients between the prospective and retrospective cohort study groups. The depression risk in the retrospective cohort study group (OR = 1.43; 95% CI: 1.30–1.58; *I*^2^ = 70.8%; Figure 3) was slightly higher than that in the prospective study cohort group (OR = 1.31; 95% CI: 1.22–1.42; *I*^2^ = 49.1%; Figure 3). Sensitivity analysis showed that none of the individual studies had reversed the pooled-effect size, which means that the results are robust (Supplementary Figure S2).

#### 3.5.2 Hearing loss type

**Figure 4:**
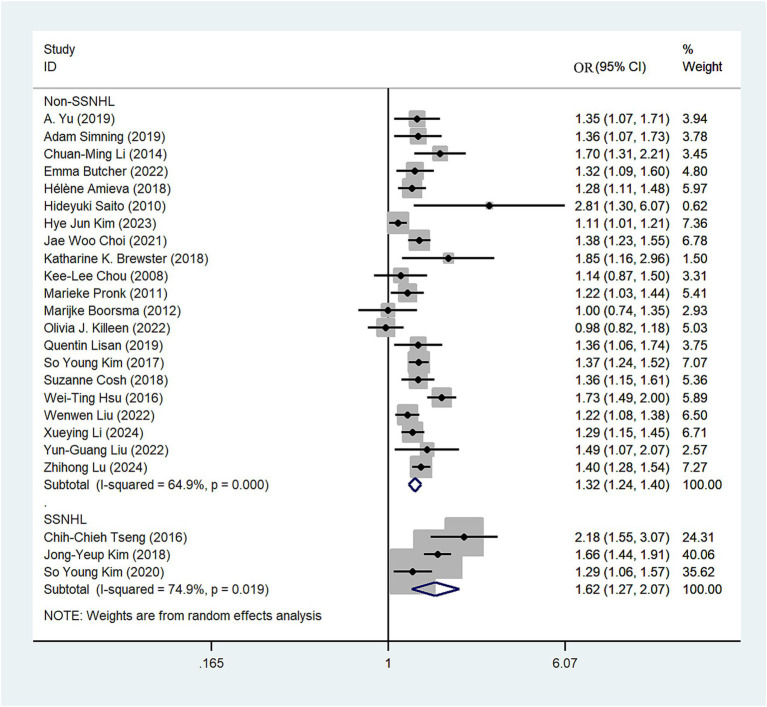
Forest plot of subgroup analysis by hearing loss type.

We classified the types of hearing loss present in the included articles, with four studies categorized under sudden sensorineural hearing loss (SSNHL). The random-effects model revealed that SSNHL significantly increased the risk of depression (OR = 1.62; 95% CI: 1.27–2.07; *I*^2^ = 74.9%; Figure 4). In the non-SSNHL group, the risk of depression was (OR = 1.32; 95% CI: 1.24–1.40; *I*^2^ = 64.9%; Figure 4). We conducted sensitivity analyses on both the SSNHL and Non-SSNHL groups, and none of the studies reversed the magnitude of the summary effect, indicating the robustness of the research findings (Supplementary Figures S3, S4).

#### 3.5.3 Follow-up years

**Figure 5:**
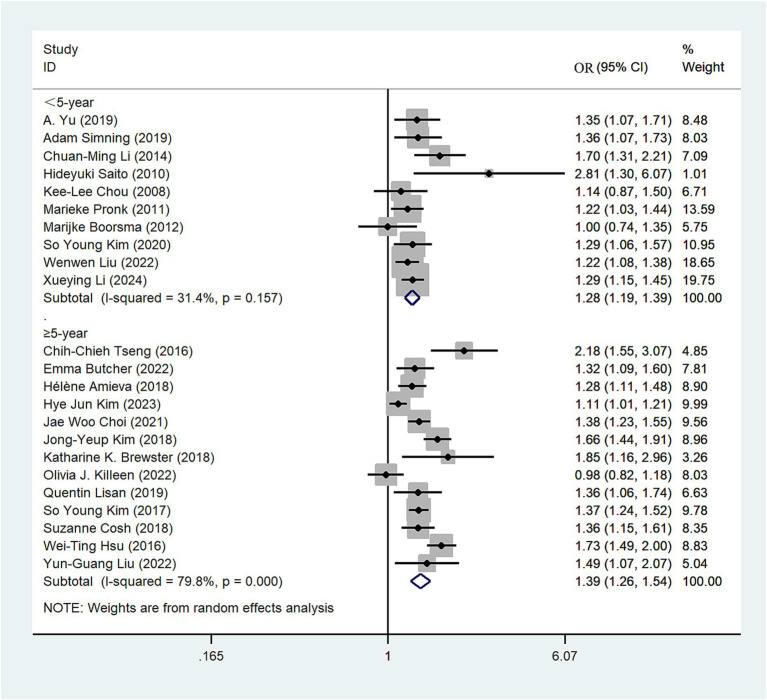
Forest plot of subgroup analysis by follow-up years.

The 23 studies (10–13, 23–37, 39–42) were stratified into two subgroups based on the duration of follow-up (<5 years, ≥5 years). Among those with a follow-up period of <5 years, individuals with hearing impairment had a 1.28-fold higher risk of depression compared to those without impairment (OR = 1.28; 95% CI: 1.19–1.39, *I*^2^ = 31.4%; Figure 5). Conversely, in the cohort with a follow-up period of ≥5 years, the risk of depression was 1.39-fold higher in individuals with hearing impairment compared to those without impairment (OR = 1.39; 95% CI: 1.26–1.54, *I*^2^ = 79.8%; Figure 5). Sensitivity analysis indicates that no single study significantly altered the magnitude of the summary effect, suggesting that the results are robust (Supplementary Figure S5).

#### 3.5.4 Age group

**Figure 6:**
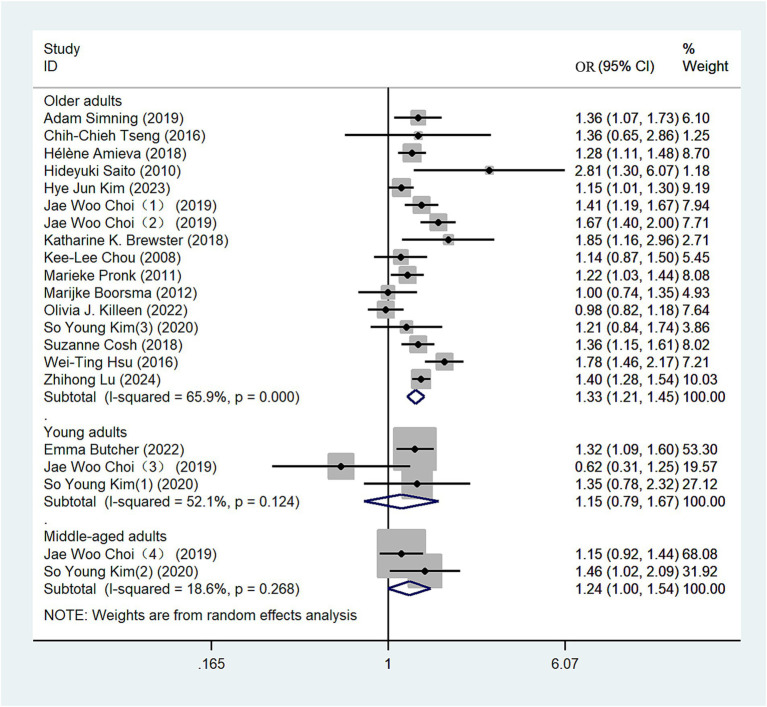
Forest plot of subgroup analysis by age group.

Data from 16 eligible studies were included in this analysis (10, 13, 23–27, 29–31, 33, 36, 38–41). We conducted subgroup analyses based on age categories: individuals aged 44 years or younger were classified as young adults, those aged 45–59 as middle-aged adults, and those aged 60 years or older as older adults. Using a random-effects model, the comprehensive meta-analysis revealed varying risks of depression among the groups: young adults (OR = 1.15, 95% CI: 0.79–1.67; *I*^2^ = 52.1%; Figure 6), middle-aged adults (OR = 1.24, 95% CI: 1.00–1.54; *I*^2^ = 18.6%; Figure 6), and older adults (OR = 1.33, 95% CI: 1.21–1.45; *I*^2^ = 65.9%; Figure 6). These findings suggest a notably high risk of depression among individuals with hearing loss in the young and middle-aged groups. Additionally, older adults demonstrate a higher risk of depression compared to both young and middle-aged adults. We conducted a sensitivity analysis, and the results remained robust (Supplementary Figure S6).

### 3.6 Publication bias

**Figure 7:**
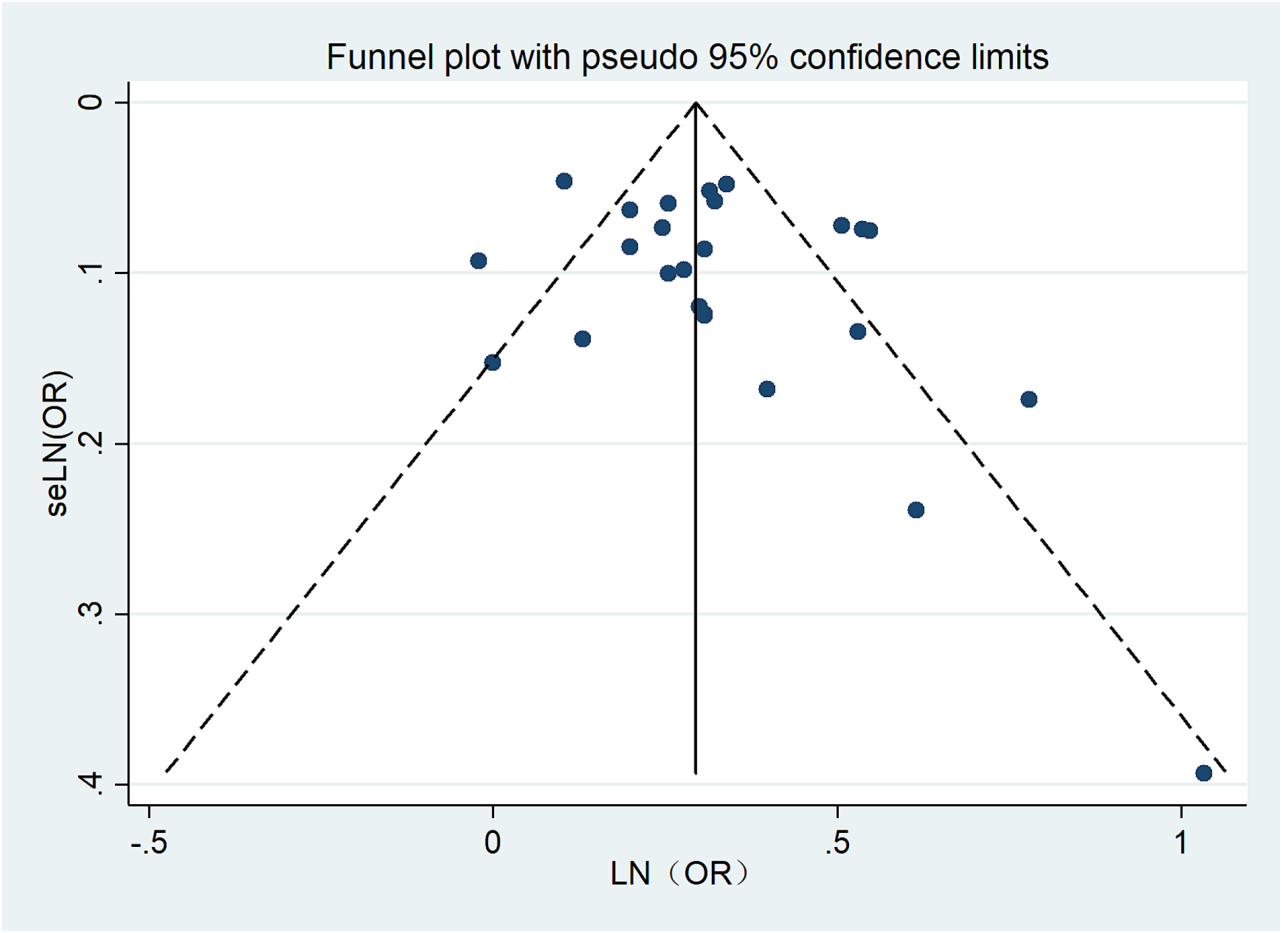
Funnel plot for publication bias.

The funnel plot for hearing loss was relatively symmetrical, indicating no publication bias. Egger’s test (*p* = 0.213) revealed no bias for hearing loss. The funnel plot is shown in Figure 7.

## 4 Discussion

### 4.1 Principal findings

This meta-analysis included 24 cohort studies covering 24,304 individuals, which provided a comprehensive evaluation on the association between HL and depression. We found a significant increase in the risk of depression among individuals with HL, with an overall 1.35-fold increase in risk, respectively, compared with non-hearing loss. Through further subgroup analysis, we found that patients with SSNHL have a higher risk of depression compared to those with non-SSNHL. Studies with a follow-up period exceeding 5 years showed a higher risk of depression among patients with hearing loss compared to those with a follow-up period of less than 5 years. Additionally, retrospective cohort studies demonstrated a higher risk of depression among patients with HL compared to prospective studies. And the risk of depression is higher in older adult patients compared to young and middle-aged patients. These findings suggest that hearing loss may be an independent risk factor for depression.

### 4.2 Comparison with previous studies

Lawrence et al.’s meta-analysis predominantly consisted of cross-sectional studies (24 studies), which, in contrast to cohort studies (11 studies), heavily emphasized associations assessed at a single time point, thus lacking longitudinal inference (43). Therefore, integrating high-quality cohort studies enables us not only to observe longitudinal associations and the chronological order of disease occurrences but also to investigate causal relationships between diseases. Building upon their initial four cohort studies (24, 25, 28, 39), we synthesized recent research to reveal new insights (10, 13, 18, 27, 29–31, 34, 37), confirming that hearing loss remains a risk factor for depression among middle-aged and young populations. Previous research by Keidser et al. (44) have also confirmed that the association between hearing loss and depression extends beyond older adults to include middle-aged individuals. This aligns with our perspective. However, our meta-analysis incorporated a larger number of high-quality cohort studies, providing a more extensive sample size. This revealed that older adults with hearing loss are at a higher risk of depression compared to younger and middle-aged individuals. Zhang et al.’s meta-analysis (20) only included patients with hearing loss assessed through pure-tone audiometry, questionnaires, or self-reports, whereas we expanded upon this foundation by incorporating insurance claims records and hospital diagnostic records (13, 25, 27, 30–32, 35, 41), thus enriching the dataset comprehensively. In contrast to Zhang et al.’s study, we extracted data specifically related to sudden sensorineural hearing loss and identified it as a risk factor for depression, addressing a previously unexplored aspect of the dataset (13, 32, 34). Both the meta-analyses by Lawrence et al. and Zhang et al. lacked investigations into follow-up periods. In contrast, our study revealed that patients with a follow-up period of ≥5 years exhibited a higher risk of depression compared to those with <5 years of follow-up. Overall, compared to previous meta-analyses, our study included a greater number of high-quality cohort studies investigating longitudinal associations between HL and depression. Additionally, we examined findings regarding the relationship between sudden sensorineural hearing loss and the risk of depression across different time frames, thereby deepening our study and addressing gaps in the existing research.

It is important to note that our assessment of different follow-up periods revealed that studies with longer follow-up found a higher risk of depression in individuals with untreated hearing loss compared to those with shorter follow-up periods. The influence of follow-up duration on disease observation depends on several factors. Generally, longer follow-up periods provide a more comprehensive understanding of disease progression, treatment effectiveness, and long-term outcomes. While short-term follow-ups are useful for initial assessments and immediate treatment responses, they may not capture the full spectrum of disease patterns or late-onset complications. In contrast, extended follow-up periods offer valuable insights into the evolution of the disease over time. A recent study on the progression of hearing loss found that, in certain subgroups, the rate of hearing loss progression could reach up to 7.1 dB per year over time (45). Additionally, a long-term follow-up study on childhood hearing loss observed that 40.3% of ears showed some degree of deterioration over a follow-up period of up to 6 years (46). In summary, the duration of follow-up may be a significant factor influencing the results of our study. Our aggregated findings indicate that the pooled OR is higher in study groups with longer follow-up periods compared to those with shorter follow-up times. It is possible that patients currently under shorter follow-up durations may face increased risks if monitored for longer periods; however, there is currently no definitive research to support this hypothesis. Additionally, our study found that the risk of depression in patients with hearing loss was higher in retrospective cohort studies than in prospective cohort studies. This discrepancy may be attributed to differences in sample selection, data collection methods, and reporting biases inherent in the two study designs. In retrospective cohort studies, case selection often relies on hospital diagnostic codes and insurance data, which may include some patients with milder early-stage conditions. Furthermore, retrospective studies tend to report positive outcomes more frequently. Consequently, the pooled OR for retrospective cohort studies is often higher than that of prospective cohort studies. It is also important to recognize that retrospective cohort studies typically have longer follow-up periods, which can be considered an advantage of this design. Therefore, future research should prioritize high-quality prospective cohort studies. Researchers designing these studies should take into account the impact of follow-up duration on disease outcomes, potentially extending the follow-up period where appropriate. To facilitate this, establishing a comprehensive national database with long-term follow-up capabilities may be necessary. Furthermore, our research expanded beyond studies focused solely on older adults to include participants across all age groups. Hearing loss can occur at any stage of life, including childhood (26, 46), though it is more common among older adults. Epidemiological studies have shown a significant increase in the prevalence of hearing problems starting around age 55 (6). Consequently, much of the existing research has concentrated on the relationship between age-related hearing loss and depression in older adults. In reality, early hearing loss is associated with delays in auditory and communicative development (15), which may contribute to social disengagement and subsequent depression during adolescence, adulthood, or even later in life. Additionally, adults with hearing loss often face communication difficulties that can impact their ability to find and maintain employment, leading to social and familial withdrawal (47). This social isolation can ultimately result in depression. Therefore, our research advocates for greater attention not only to hearing loss in older adults but also to its effects in younger and middle-aged individuals, and even in children. Early awareness of hearing deterioration is crucial for understanding its potential impacts on early auditory and language development, as well as on other areas such as social and academic functions (46). We urge future researchers to expand their focus beyond older adults with hearing loss to include younger, middle-aged, and even pediatric populations, despite the challenges in assessing hearing loss and depression in children (48). Early intervention for hearing impairment should be considered across all age groups, whether individuals are children, adolescents, or middle-aged adults.

### 4.3 Interpretation of findings

To date, several hypotheses exist regarding the risk mechanisms linking HL to the occurrence of depression. For instance, negative emotions associated with hearing impairment, such as feelings of loneliness or social isolation, may contribute to an increased risk of subsequent depression (49, 50). Furthermore, research suggests that hearing deficits could potentially lead to neural cognitive control network atrophy through reduced peripheral input, thereby escalating the risk of depression (17, 40). In a recent study, HL was found to be associated with smaller brain volumes, including total cranial and gray matter volumes, as well as auditory cortex and limbic system volumes (46). This implies that hearing loss might serve as an indicator accelerating brain atrophy. In the sole available neuroimaging study exploring the relationship between hearing impairment and emotion processing, Husain et al. discovered that hearing-impaired individuals exhibited diminished amygdala and parahippocampal responses to emotionally charged sounds from the International Affective Digital Sounds database. Additionally, they showed prolonged reaction times to pleasant and unpleasant (but not neutral) auditory stimuli (51). This may be one of the mechanisms contributing to depression following HL. The findings from our meta-analysis offer valuable insights for both clinicians and individuals with HL. Firstly, they emphasize the importance of advocating for effective HL management. Secondly, by acknowledging the uncertainty regarding the potential link between HL and depression, there is an opportunity to alleviate the psychological burden associated with HL.

### 4.4 Strengths and limitations

Given the lack of previous meta-analyses, we conducted an extensive analysis of the relationship between hearing loss and depression, examining various factors including age groups, cohort types, follow-up durations, and types of hearing impairment. Our findings revealed differing results across subgroups, yet consistently highlighted a significant correlation between hearing loss and depression. Our research identified that individuals across different age brackets—young, middle-aged, and older adults—with hearing impairment are at increased risk of depression, underscoring the importance of preventive measures, particularly early interventions for younger populations. Despite these insights, our meta-analysis inevitably faces certain limitations. Observational studies inherently carry the risk of confounding biases, despite our efforts to select results from studies that control for confounders to bolster the robustness of our findings. Unlike previous meta-analyses, we exclusively included cohort studies to observe the temporal sequence between hearing loss and depression onset, enhancing the longitudinal and practical relevance of our results while excluding cross-sectional studies incapable of analyzing the temporal order of events. However, our inclusion of a substantial number of retrospective cohorts means that recall biases cannot be entirely eliminated. Therefore, future research should prioritize more rigorous prospective cohort designs to address this shortfall and.

## 5 Conclusion

Our updated meta-analysis revealed a heightened risk of depression associated with HL. However, significant heterogeneity was observed, and the underlying mechanism remains unclear. Nonetheless, additional studies are required to validate the pathophysiological mechanisms driving this phenomenon.

## Data Availability

The raw data supporting the conclusions of this article will be made available by the authors, without undue reservation.
